# Lipoprotein(a) as an Independent Predictor of Elevated SYNTAX Score

**DOI:** 10.3390/jcm13237109

**Published:** 2024-11-24

**Authors:** Monika Kozieł-Siołkowska, Katarzyna Mitręga, Tomasz Podolecki, Anna Olma, Zbigniew Kalarus, Witold Streb

**Affiliations:** 1 1st Department of Cardiology and Angiology, Silesian Center for Heart Diseases, 41-800 Zabrze, Poland; kozielmonika@poczta.fm (M.K.-S.); kas-k2@o2.pl (K.M.); a_wizner@wp.pl (A.O.); 2 Department of Cardiology, Congenital Heart Diseases and Electrotherapy, Faculty of Medical Sciences in Zabrze, Medical University of Silesia, 40-055 Katowice, Poland; tomekpod@interia.pl (T.P.); zbigniew.kalarus@kalmet.com.pl (Z.K.)

**Keywords:** atherosclerotic cardiovascular disease, acute myocardial infarction, coronary artery atherosclerosis, lipoprotein(a), SYNTAX score

## Abstract

**Background/Objectives:** Increased lipoprotein(a) [Lp(a)] level is associated with elevated possibility of atherosclerosis progression. SYNTAX score enables to grade the anatomy of coronary arteries. To identify the impact of increased Lp(a) level on SYNTAX score in individuals with acute myocardial infarction (AMI). **Methods:** In our analysis, we enrolled 173 consecutive adult patients hospitalized for AMI in a tertiary cardiology center from December 2022 to August 2023. Patient characteristics were compared for patients with SYNTAX score ≥ 23 (64 patients) and SYNTAX score < 23 (109 patients). The SYNTAX score was estimated based on the results of coronary angiography. Logistic regression analyses were performed to evaluate the factors associated with SYNTAX score. **Results:** Individuals with the SYNTAX score ≥ 23 were more likely to have arterial hypertension, diabetes mellitus, significant stenosis in the left main coronary artery, and higher Lp(a) levels than those with SYNTAX < 23 (all *p* < 0.05). On univariate analysis, age (OR 1.05, 95% CI 1.02–1.08, *p* = 0.001), Lp(a) levels (OR 1.04, 95% CI 1.01–1.06, *p* = 0.001), and arterial hypertension (OR 2.69, 95% CI 1.26–5.74, *p* = 0.011) were associated with SYNTAX score ≥ 23. Multivariable determinants of SYNTAX score ≥ 23 were as follows: Lp(a) levels (OR 1.03, 95% CI 1.01–1.08, *p* = 0.029), and age (OR 1.04, 95% CI 1.01–1.07, *p* = 0.005). The cut-off value for Lp(a) 166.16 nmol/L identifies patients with SYNTAX score ≥ 23 with 97% sensitivity and 44% specificity (area under curve 0.78, *p* < 0.001). **Conclusions:** Elevated Lp(a) concentration is associated with a higher SYNTAX score. A cut-off value of Lp(a) above 166.16 nmol/L allows us to identify subjects with SYNTAX score ≥ 23 with good specificity and sensitivity.

## 1. Background

Lipoprotein(a) or Lp(a) is a form of low-density lipoprotein (LDL) molecule consisting of an apolipoprotein(a) component covalently connected to its apolipoprotein B (ApoB) part [1]. The level of Lp(a) in serum is genetically established and consistent in particular patients, and it remains unaltered by diet or exercise [2]. It accumulates within the walls of arteries, and then it may lead to an increasing risk of atherosclerotic cardiovascular disease (ASCVD) involving atherosclerotic stenosis of arteries, myocardial infarction, and aortic valve stenosis. Furthermore, this particle induces vascular inflammation, atherogenesis, calcification, and thrombosis [3]. It is mostly a monogenic cardiovascular risk factor, with approximately 70% to 90% of interindividual diversity in levels that are genetically determined [4].

The Mendelian randomization approach confirmed that the causal effect of Lp(a) on the risk of ASCVD is proportional to the absolute fluctuations of Lp(a) levels in plasma. Moreover, individuals with very high Lp(a) levels > 180 mg/dL (>430 nmol/L) tend to have an elevated risk of ASCVD during their whole life, comparable to that of individuals with confirmed heterozygous familial (HeFH). Since approximately 90% of individual Lp(a) levels are inherited, a very high Lp(a) level may constitute a newly inherited lipid abnormality, which is associated with severely increased lifetime risk of ASCVD and is two-fold more frequent than HeFH [5].

Knowledge of the existence of an increased Lp(a) level is crucial because an elevated Lp(a) level increases the risk of ASCVD and may affect clinical decision-making concerning risk management.

Increased Lp(a) level is associated with the development and progression of atherosclerotic cardiovascular disease. Individuals with a history of AMI and elevated Lp(a) should be medicated with intensive statin therapy and adjunctive medications as initial therapy. Available data report that PCSK9 inhibitors also decrease Lp(a) plasma levels. Moreover, the evidence in favor of screening for Lp(a) is the strongest for patients with a family or personal history of ASCVD, with consideration of cascade screening in appropriate individuals.

The SYNTAX score was established as an angiographic stratification tool to assess the complexity of coronary lesions in individuals with multivessel coronary artery disease (CAD) or left main coronary artery. It also helped clinicians in deciding the optimal revascularization procedure [6].

## 2. Objectives

The aim of our study was to identify the impact of elevated Lp(a) on SYNTAX in individuals diagnosed with AMI.

## 3. Materials and Methods

This study group comprised 173 consecutive adult patients with confirmed AMI (ST-elevation myocardial infarction or non-ST-elevation myocardial infarction) admitted to the tertiary cardiac center (Department of Cardiology and Electrotherapy of Medical University of Silesia, Silesian Centre for Heart Diseases in Zabrze) between December 2022 and August 2023. Inclusion criteria included the following: age ≥ 18 years, AMI, measured Lp(a) levels, and performed coronary angiography.

Exclusion criteria included the following: individuals aged < 18 years or those who refused enrollment in our trial.

AMI was diagnosed based on the current guidelines and included patients with confirmed ST-elevation myocardial infarction (STEMI) or non-ST-elevation myocardial infarction (NSTEMI) [6].

Lipoprotein(a) levels were measured on admission using the Atellica Solution CH 930 chemical analyzer utilizing the immunoturbidimetry method.

Patients were classified into two groups according to their SYNTAX score: group with SYNTAX score ≥ 23 (n = 64), and group with SYNTAX score < 23 (n = 109).

The SYNTAX score was calculated based on the results of coronary angiography.

Our study was conducted in accordance with international standards, among others, the Declaration of Helsinki.

### Statistical Analysis

We presented categorical variables as absolute frequencies and percentages. We calculated normality of distribution using the Shapiro–Wilk test. The quantitative variables that did not have a normal distribution were shown as medians and interquartile ranges. Quantitative variables that were normally distributed were expressed as means with standard deviations. Group comparisons were analyzed using the Student’s *t*-test (for variables that were normally distributed) or the Mann–Whitney test (used for non-normally distributed variables). Fisher’s test was used to analyze qualitative data to compare groups. The descriptive analysis involved baseline characteristics of patients. A two-sided *p*-value of less than 0.05 was defined as statistically significant. Patient baseline characteristics were compared for individuals with SYNTAX score ≥ 23 and those with SYNTAX score < 23. Logistic regression analyses were prepared to evaluate the factors associated with the SYNTAX score. The variables with statistically significant association on univariate logistic regression analysis were put into multivariable logistic regression models. The results were presented as a hazard ratio (HR) with a 95% confidence interval (CI). To find the cut-off value for Lp(a) associated with SYNTAX score ≥ 23, ROC analysis was performed. The Youden index was used to determine the best cut-off.

## 4. Results

Individuals with SYNTAX score ≥ 23 were more likely to be older than those with SYNTAX score < 23 (Table 1). They were more likely to have arterial hypertension, diabetes mellitus, and significant stenosis in the left main coronary artery. They also had a greater number of coronary arteries with significant stenoses than those with SYNTAX score < 23 (Table 1).

Both analyzed groups did not differ according to total cholesterol, HDL, LDL, and non-HDL. However, Lp(a) concentrations were more than 3 times greater in the SYNTAX score ≥ 23 groups (49.1 (14.0, 175.0) vs. 15.5 (8.1, 37.3), *p* < 0.05), Table 2.

On univariate analysis, SYNTAX score ≥ 23 was significantly associated with Lp(a) levels (OR 1.04, 95% CI 1.01–1.06, *p* = 0.01), age (OR 1.05, 95% CI 1.02–1.08, *p* = 0.001), and arterial hypertension (OR 2.69, 95% CI 1.26–5.74, *p* = 0.011), Table 3.

The multivariable analysis allows us to establish that SYNTAX score ≥ 23 was significantly associated only with Lp(a) levels (OR 1.03, 95% CI 1.01–1.08, *p* = 0.029), and age (OR 1.04, 95% CI 1.01–1.07, *p* = 0.005), Table 3.

Receiver-operating analysis performed for the Lp(a) indicated a cut-off value of 166.16 nmol/L as a good marker of SYNTAX score ≥ 23 (area under curve 0.78, *p* < 0.001, sensitivity 97%, specificity 44%), Figure S1, Supplementary Materials.

## 5. Discussion

This snapshot survey presents a contemporary insight into factors associated with elevated SYNTAX score in real-world individuals with AMI.

Our findings demonstrate that factors independently associated with SYNTAX score ≥ 23 were age and levels of Lp(a).

Xu et al. demonstrated that increased Lp(a) level was an independent predictor of a SYNTAX score ≥ 23 only in patients with LDL-C ≥ 100 mg/dl and chronic coronary syndromes [7]. The positive correlation between SYNTAX score and Lp(a) levels was consistent with the results of our study. In another study, individuals with premature coronary artery disease and elevated Lp(a) levels had significantly more complex coronary atherosclerotic disease as calculated by greater SYNTAX scores [8].

Elevated Lp(a) concentration is associated with higher SYNTAX score in a few studies with the Australian, Indian, and Chinese populations, similar to our results [8,9,10,11]. This association is also present in the Polish population.

Leistner et al. demonstrated that patients with elevated Lp(a) levels had a greater number of atherosclerotic plagues, a higher SYNTAX-I score, and a higher percentage of diffusely narrowed vessels and chronic total occlusions than individuals with normal Lp(a) levels [12].

Data on the link between elevated Lp(a) levels and the extent and severity of coronary artery atherosclerosis are generally scarce. Some clinical studies have presented that increased levels of Lp(a) are associated with the progression of coronary artery atherosclerosis [13,14,15]. However, there is limited data on the usefulness of Lp(a) in predicting the advancement of atherosclerosis in coronary arteries in individuals with AMI. This study therefore complements the Lp(a) data collected in clinical practice and reduces gaps in the knowledge.

In some studies, elevated Lp(a) levels were associated with a higher probability of advanced coronary atherosclerosis and AMI, especially in Latin Americans and South Asians [16,17,18].

In the Chinese population, increased levels of Lp(a) in plasma may contribute to the exacerbation and progression of atherosclerosis [19]. A number of observational studies have reported that elevated Lp(a) levels might be the factors linked with the advancement of CAD in the Chinese population [20,21].

Several studies demonstrated a link between increased levels of Lp(a) and serial plague progression in previous angiographic and tomographic studies [22,23,24]. In one study, serial intravascular ultrasound revealed a correlation between increased Lp(a) levels and plaque progression in the left main coronary arteries [25]. Moreover, high Lp(a) levels are associated with coronary stenosis progression in a short time in individuals with AMI, especially the new lesion revascularization [26]. Patients with very high (>100 mg/dL) Lp(a) levels and AMI had an increased SYNTAX score documented at coronary angiography and had more often multivessel disease when compared to those with normal (<50 mg/dL) and increased (>50 mg/dL) Lp(a) levels [27]. Individuals with very high Lp(a) levels and AMI had significantly more severe CAD burden, even then adjusted for classical risk factors and age [28]. The rapid progression of coronary artery disease in patients with increased Lp(a) levels may be related to an interference with thrombolysis through the partial structural similarity of Lp(a) and plasminogen [13].

In the present analysis, we have demonstrated that increased Lp(a) levels were independent predictors of the severity and complexity of atherosclerosis in patients with AMI. Individuals with higher SYNTAX score can be identified based on Lp(a) levels. These findings are comparable to those presented in previous studies [8,28].

Controlling risk factors, especially emerging ones like Lp(a) tends to be crucial in patients with AMI. However, this is important not only in those with elevated SYNTAX score or obstructive CAD but also in the myocardial infarction with non-obstructed coronary arteries (MINOCA) population, where strict lipid control, including LDL and Lp(a), is critical due to the high probability of reinfarction and plaque progression often demanding revascularization, as recently demonstrated [29].

## 6. Limitations of This Study

Our study has some limitations that should be listed. Firstly, this is a single-center, observational analysis from a tertiary care university hospital based on Caucasian race. Secondly, this study sample is relatively small. Moreover, increased Lp(a) levels have also been described in some inflammatory diseases, such as chronic kidney disease, pulmonary arterial hypertension, systemic lupus erythematosus, and rheumatoid arthritis [30,31,32,33]. Furthermore, limitations of the SYNTAX score are inter-observer variability in its calculations. The above-mentioned score quantifies obstruction and does not focus on the plague burden [34,35].

## 7. Conclusions

Elevated Lp(a) concentration is associated with a higher SYNTAX score. A cut-off value of Lp(a) above 166.16 nmol/L allows us to identify subjects with SYNTAX score ≥ 23 with good specificity and sensitivity. Taking into account the single-center observational study limitations, more evidence is needed.

## Figures and Tables

**Table 1:** Baseline characteristics of individuals with AMI according to SYNTAX score.

Variable	Whole Population n = 173	Pts with SYNTAX Score ≥ 23 n = 64	Pts with SYNTAX Score < 23 n = 109	*p*-Value
Male, gender, n (%)	113 (65.3)	40 (62.5)	73 (67.0)	0.553
Age, years, median (Q1, Q3)	66.5 (57.0, 74.0)	69.0 (64.0, 78.0)	64.0 (54.0, 72.5)	0.001
Nicotinism, n (%)	47 (27.2)	15 (23.4)	32 (29.4)	0.401
Arterial hypertension, n (%)	121 (69.9)	52 (81.3)	69 (63.3)	0.008
Diabetes mellitus, n (%)	59 (34.1)	28 (43.8)	31 (28.4)	0.033
Chronic kidney disease, n (%)	18 (10.4)	9 (14.1)	9 (8.3)	0.216
NSTEMI, n (%)	77 (44.5)	34 (53.1)	43 (39.4)	0.081
STEMI, n (%)	95 (54.9)	29 (45.3)	66 (60.6)	0.052
Significant stenosis in LM, n (%)	12 (6.9)	10 (15.6)	2 (1.8)	<0.001
Number of coronary arteries with significant stenoses, median (Q1, Q3)	2.0 (1.0, 3.0)	3.0 (2.0, 4.0)	1.0 (1.0, 2.0)	<0.001

AMI, acute myocardial infarction, LM, left main, NSTEMI, non-ST-elevation myocardial infarction, Q1, the lower quartile, Q3, the upper quartile, STEMI, ST-elevation myocardial infarction.

**Table 2:** Baseline laboratory measurements in individuals with AMI based on SYNTAX score.

Variable	Whole Population n = 173	Pts with SYNTAX Score ≥ 23 n = 64	Pts with SYNTAX Score < 23 n = 109	*p*-Value
TC, mmol/L, median (Q1, Q3)	4.9 (4.1, 5.8)	4.7 (3.9, 5.7)	5.2 (4.1, 5.9)	0.105
LDL-C, mmol/L, mean (SD)	3.1 (2.3, 3.9)	2.9 (2.2, 3.9)	3.2 (2.3, 4.1)	0.197
HDL-C, mmol/L, mmol/L, median (Q1, Q3)	3.1 (2.3, 3.9)	1.2 (0.9, 1.4)	1.2 (0.9, 1.5)	0.153
Non-HDL, mmol/L, median (Q1, Q3)	3.7 (2.7, 4.6)	3.5 (2.6, 4.7)	4.0 (2.7, 4.6)	0.160
TGs, mmol/L, median (Q1, Q3)	1.3 (0.8, 1.8)	1.3 (0.8, 1.6)	1.2 (0.8, 1.9)	0.411
Lp(a), nmol/L, median (Q1, Q3)	20.7 (10.2, 75.0)	49.1 (14.0, 175.0)	15.5 (8.1, 37.3)	<0.001
Creatinine, μmol/L, median (Q1, Q3)	90.0 (75.0, 112.0)	93.0 (76.0, 116.0)	89.0 (75.0, 110)	0.462
CRP, mg/L, median (Q1, Q3)	4.9 (1.3, 11.7)	7.0 (1.9, 16.6)	3.7 (1.2, 9.3)	0.075

CRP, C-reactive protein, HDL-C, high-density lipoprotein cholesterol, LDL-C, low-density lipoprotein cholesterol, Lp(a), lipoprotein(a), non-HDL, non-high-density lipoprotein cholesterol, Q1, the lower quartile, Q3, the upper quartile, SD, standard deviation, TC, total cholesterol, TGs, triglycerides.

**Table 3:** Factors associated with SYNTAX score ≥ 23.

	Univariate Analysis			Multivariate Analysis		
Variable	OR	95% CI	*p*-Value	OR	95% CI	*p*-Value
Age	1.05	1.02–1.08	0.001	1.04	1.01–1.07	0.005
Nicotinism	0.77	0.38–1.57	0.467			
Gender, male	0.85	0.44–1.65	0.634			
Lipoprotein(a), nmol/L	1.04	1.01–1.06	0.001	1.03	1.01–1.08	0.029
T chol (mmol/L)	0.80	0.63–1.02	0.074			
TG (mmol/L)	0.88	0.65–1.20	0.416			
Corrected LDL (mmol/L)	0.82	0.62–1.08	0.152			
HDL (mmol/L)	0.56	0.27–1.16	0.119			
Non-HDL (mmol/L)	0.82	0.65–1.05	0.123			
HbA1c (%)	1.04	0.81–1.33	0.759			
Creatinine (μmol/L)	1.01	0.99–117	0.485			
CRP (mg/L)	1.03	0.99–1.02	0.075			
LVEF (%)	1.01	0.98–1.03	0.628			
Arterial hypertension	2.69	1.26–5.74	0.011			
Diabetes	1.72	0.89–3.32	0.107			
Chronic kidney disease	1.71	0.62–4.69	0.298			

CI, confidence interval, LVEF, left ventricular ejection fraction, OR, odds ratio

## Data Availability

Not applicable.

## Supplementary Materials

The following supporting information can be downloaded at https://www.mdpi.com/article/10.3390/jcm13237109/s1. Figure S1. Receiver-operating curves.
