# Supplementary figures and images for: Lipoprotein(a) as an Independent Predictor of Elevated SYNTAX Score
Source: J Clin Med. 2024 Nov 24;13(23):7109. doi: 10.3390/jcm13237109 (PMC11642446; doi:10.3390/jcm13237109)

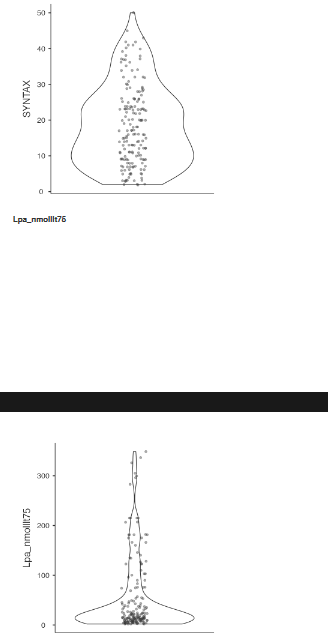

Supplement: Supplementary file 1 [file jcm-13-07109-s001.zip › jcm-3297339-supplementary.png]
